# Supplementary material for: The adipocyte hormone leptin sets the emergence of hippocampal inhibition in mice
Source: eLife. 2018 Aug 14;7:e36726. doi: 10.7554/eLife.36726 (PMC6112852; doi:10.7554/eLife.36726)
Supplement: Transparent reporting form [file elife-36726-transrepform.pdf]

eLIFE
1st Floor
24 Hills Road
Cambridge CB2 1JP, UK
P 01223 855340
W elifesciences.org
T @elife

eLife's transparent reporting form

We encourage authors to provide detailed information *within their submission* to facilitate the interpretation and replication of experiments. Authors can upload supporting documentation to indicate the use of appropriate reporting guidelines for health-related research (see EQUATOR Network), life science research (see the BioSharing Information Resource), or the ARRIVE guidelines for reporting work involving animal research. Where applicable, authors should refer to any relevant reporting standards documents in this form.

If you have any questions, please consult our Journal Policies and/or contact us: editorial@elifesciences.org.

Sample-size estimation

- You should state whether an appropriate sample size was computed when the study was being designed
- You should state the statistical method of sample size computation and any required assumptions
- If no explicit power analysis was used, you should describe how you decided what sample (replicate) size (number) to use

Please outline where this information can be found within the submission (e.g., sections or figure legends), or explain why this information doesn't apply to your submission:

No statistical methods were used to predetermine sample sizes when the study was designed. We decided the sample size based on our previous studies with similar experiments.

The one way ANOVA followed by a Tukey's *post hoc* test was used for multiple comparisons between experimental conditions. The one way ANOVA was used to analyze difference between two individual groups. A two-tailed paired Student's *t*-test was used to analyze differences within one group across conditions.

This information can be found in the material and method, section statistic.

Sample size (number of animal used, number of cell cultures prepared, and number of cell recorded) is indicated in each figure. P values and statistical test used are indicated in each figure legend.

Replicates

- You should report how often each experiment was performed
- You should include a definition of biological versus technical replication
- The data obtained should be provided and sufficient information should be provided to indicate the number of independent biological and/or technical replicates
- If you encountered any outliers, you should describe how these were handled
- Criteria for exclusion/inclusion of data should be clearly stated
- High-throughput sequence data should be uploaded before submission, with a private link for reviewers provided (these are available from both GEO and ArrayExpress)

Please outline where this information can be found within the submission (e.g., sections or figure legends), or explain why this information doesn't apply to your submission:

All data sets that are presented in our manuscript were conducted in different neuronal cultures or acute hippocampal slices obtained from at least three different animals (biological replicate). This is stated in the material and method section of our manuscript. Details on the number of mice, neuronal cultures and cells are indicated in the figures.

No outliers are identified or removed from our analyses.

We used standard criteria (cell input resistance, resting membrane potential at zero current, baseline stability) for ascertaining the quality of the electrophysiological recordings.

Statistical reporting

- Statistical analysis methods should be described and justified
- Raw data should be presented in figures whenever informative to do so (typically when N per group is less than 10)
- For each experiment, you should identify the statistical tests used, exact values of N, definitions of center, methods of multiple test correction, and dispersion and precision measures (e.g., mean, median, SD, SEM, confidence intervals; and, for the major substantive results, a measure of effect size (e.g., Pearson's r, Cohen's d)
- Report exact p-values wherever possible alongside the summary statistics and 95% confidence intervals. These should be reported for all key questions and not only when the p-value is less than 0.05.

Please outline where this information can be found within the submission (e.g., sections or figure legends), or explain why this information doesn't apply to your submission:

All data analyses were performed using mainly nonparametric statistical tools due to the size of the samples (http://astatsa.com/).
The one way ANOVA followed by a Tukey's *post hoc* test was used for multiple comparisons between experimental conditions. A two-tailed unpaired Student's *t*-test was used to analyze difference between two individual groups. A two-tailed paired Student's *t*-test was used to analyze differences within one group across conditions. The level of significance was set at a P-value less than 0.05. Appropriate statistical tests were conducted depending on the experiment and are indicated in the figure legends. More specifically, statistical tests are presented as following:

Figure 1B: one way ANOVA followed by a Tukey's *post hoc* test.
Figure 2:
B and F) one way ANOVA followed by a Tukey's *post hoc* test.
D) two-tailed unpaired Student's *t*-test
Figure 3:
B and D) two-tailed paired Student's *t*-test and two-tailed unpaired Student's *t*-test.
E) one way ANOVA followed by a Tukey's *post hoc* test
F) two-tailed unpaired Student's *t*-test.
Figure 4:
B) two-tailed paired Student's *t*-test and two-tailed unpaired Student's *t*-test.
D and F) two-tailed unpaired Student's *t*-test.
Supplementary figure 1:
A, B, C and D) one way ANOVA followed by a Tukey's *post hoc* test.

(For large datasets, or papers with a very large number of statistical tests, you may upload a single table file with tests, Ns, etc., with reference to sections in the manuscript.)

Group allocation

- Indicate how samples were allocated into experimental groups (in the case of clinical studies, please specify allocation to treatment method); if randomization was used, please also state if restricted randomization was applied
- Indicate if masking was used during group allocation, data collection and/or data analysis

Please outline where this information can be found within the submission (e.g., sections or figure legends), or explain why this information doesn't apply to your submission:

For the experiments conducted on db/db, ob/ob, and wt mice allocation was based on animal genotype.

For the experiments performed on db/db, ob/ob, and wt, as well as on sham, leptin-treated, normal diet offspring, and diet-induced obesity offspring, the persons performing experiments and analyzing the data were blinded to the genotype of the mice.

This information can be found in the material and method, statistics section.

Additional data files ("source data")

- We encourage you to upload relevant additional data files, such as numerical data that are represented as a graph in a figure, or as a summary table
- Where provided, these should be in the most useful format, and they can be uploaded as "Source data" files linked to a main figure or table
- Include model definition files including the full list of parameters used
- Include code used for data analysis (e.g., R, MatLab)
- Avoid stating that data files are "available upon request"

Please indicate the figures or tables for which source data files have been provided:

The numerical data for Figures 1, 2, 3 and 4, for the supplementary figure 1 and for data not shown are given in the source data files, so that "Figure X-data source" file corresponds to the numerical data of Figure X.

eLife Sciences Publications, Ltd is a limited liability non-profit non-stock corporation incorporated in the State of Delaware, USA, with company number 5030732, and is registered in the UK with company number FC030576 and branch number BR015634 at the address 1st Floor, 24 Hills Road, Cambridge CB2 1JP | August 2014
